# Microbiota and Cyanotoxin Content of Retail Spirulina Supplements and Spirulina Supplemented Foods

**DOI:** 10.3390/microorganisms11051175

**Published:** 2023-04-30

**Authors:** Jonathan Rhoades, Stamatia Fotiadou, Georgia Paschalidou, Theodoti Papadimitriou, Avelino Álvarez Ordóñez, Konstantinos Kormas, Elisabeth Vardaka, Eleni Likotrafiti

**Affiliations:** 1 Laboratory of Food Microbiology, Department of Food Science and Technology, International Hellenic University, 57400 Thessaloniki, Greece; 2 Department of Ichthyology and Aquatic Environment, University of Thessaly, 38446 Volos, Greece; 3 Department of Food Hygiene and Technology, Universidad de León, 24004 León, Spain; 4 Agricultural Development Institiute, University Research and Innovation Centre “IASON”, Argonafton & Filellinon, 38221 Volos, Greece; 5 Department of Nutritional Sciences and Dietetics, International Hellenic University, 57400 Thessaloniki, Greece

**Keywords:** *Arthrospira*, spirulina, microcystin, cyanotoxins, microalgae, cyanobacteria, *Bacillus cereus*, bacteria, 16S rRNA sequencing, MALDI-TOF

## Abstract

Cyanobacterial biomass such as spirulina (*Arthrospira* spp.) is widely available as a food supplement and can also be added to foods as a nutritionally beneficial ingredient. Spirulina is often produced in open ponds, which are vulnerable to contamination by various microorganisms, including some toxin-producing cyanobacteria. This study examined the microbial population of commercially available spirulina products including for the presence of cyanobacterial toxins. Five products (two supplements, three foods) were examined. The microbial populations were determined by culture methods, followed by identification of isolates using matrix-assisted laser desorption ionization-time of flight mass spectrometry (MALDI-TOF), and by 16S rRNA amplicon sequencing of the products themselves and of the total growth on the enumeration plates. Toxin analysis was carried out by enzyme-linked immunosorbent assay (ELISA). Several potentially pathogenic bacteria were detected in the products, including *Bacillus cereus* and *Klebsiella pneumoniae*. Microcystin toxins were detected in all the products at levels that could lead to consumers exceeding their recommended daily limits. Substantial differences were observed in the identifications obtained using amplicon sequencing and MALDI-TOF, particularly between closely related *Bacillus* spp. The study showed that there are microbiological safety issues associated with commercial spirulina products that should be addressed, and these are most likely associated with the normal means of production in open ponds.

## 1. Introduction

Cyanobacteria, sometimes referred as cyanophytes or blue-green algae, are a large and diverse phylum of photolithotrophic prokaryotic microalgae that are ubiquitous in fresh water and marine environments. Some species produce toxins, and excessive growth of these bacteria in water bodies poses health risks to humans and animals. Other species can be harvested as a food source or dietary supplement, a practice that is known to date back to at least the 16th century [1]. Today, the two most important microalgae genera that are harvested for human consumption are the cyanobacterium *Arthrospira* and the eukaryotic single-celled green alga *Chlorella* [2]. The genus *Arthrospira* consists of around thirty-five species, of which two, *A. platensis* and *A. maxima*, are the most important in commercial production [1]. They are collectively and informally known as ‘spirulina’. However, this term is potentially confusing, as *Spirulina* is also a separate genus of cyanobacteria to which these species used to belong to before being reclassified in 1989 [3]. Throughout this manuscript, ‘spirulina’ (without italics) refers to harvested *Arthrospira* spp. biomass.

*Arthrospira* spp. are found throughout the world in fresh and saline waters. They are alkaline tolerant, which can give them a competitive advantage in lakes with a high pH [4]. They can be cultured in a variety of ways including closed photobioreactor systems, but the most common methods are through natural or artificial ponds, or raceways that are open to the environment. These require constant agitation to ensure that all cells receive sufficient light. Optimal conditions for *Arthrospira* spp. are 35 °C and a pH of 9–10 (usually adjusted with bicarbonate in the artificial systems) [5].

Once harvested, the spirulina biomass is dried and then usually powdered or compressed into tablets or bricks. It is then sold either by itself as a dietary supplement or added to various foods for consumption by humans or animals. Common examples of foods containing spirulina are cereal bars, biscuits and crackers, spreads, soups, and pasta. There are many reported nutritional benefits associated with spirulina. It has a high, if somewhat variable, protein content (normally within the range of 57–70% of dry weight), beneficial fatty acids such as γ-linoleic and palmitic acid, vitamins and vitamin precursors (notably B_12_, astaxanthin, zeaxanthin and β-carotene), and minerals (particularly iron, potassium, calcium, zinc and selenium) [1,6]. Spirulina has been shown in clinical trials to exhibit pharmaceutical properties, as it can lower blood cholesterol in some patients [7] and reduce the intensity of allergic reactions such as rhinitis [8,9]. There is also evidence of antiviral and anticancer activity [3]. Some of these beneficial activities may be attributable to the spirulina correcting nutrient deficiencies rather than any active mechanism.

Comprehensive clinical trials examining the safety of spirulina for humans are lacking. Despite this, the long history of spirulina consumption by people, together with ample data from animal feeding trials, has led to the acceptance of *Arthrospira* spp. As being safe for human consumption [3]. However, there are potential hazards associated with the usual means of production of spirulina in open ponds. Such systems are vulnerable to contamination with bacteria from animals, soil, and lake waters, including potential pathogens [10]. It is also possible that other, potentially toxin-producing, species of cyanobacteria may grow together with the desired *Arthrospira* spp. In a study by Vardaka et al. [11], 454 pyrosequencing analysis of 31 spirulina supplements available on the Greek market detected an extremely diverse microbiota of other cyanobacteria and heterotrophic bacteria. Eighteen of the thirty-one products contained from one to five additional cyanobacterial species, while >100 operational taxonomic units (OTUs) of heterotrophic bacteria were detected in ten samples.

The aim of the current work was to investigate the microbial population of spirulina supplements and foods of which spirulina is an ingredient. Rather than conducting a survey of many samples, the objective was to examine a few samples in detail using culture-dependent and -independent methodologies in order to detect potentially harmful, viable bacteria in these products. The microbial groups selected for the culture-dependent analyses were chosen to represent the common spoilage organisms in most foods, including spores that can survive heat processing and drying, pathogens and indicators that are associated with fecal contamination (of the open ponds), the handling of the products, and the aquatic/marine environment. In addition to obtaining information about the products themselves, the experimental programme was also intended to compare the different methods for the isolation and identification of foodborne bacteria.

## 2. Materials and Methods

### 2.1. Foods and Spirulina Dietary Supplements Examined

Two spirulina dietary supplements, produced in Greece by different manufacturers and containing only dried *Arthrospira* biomass, were examined. One was in tablet form and the other in powder form. Three foods to which spirulina had been added by the manufacturer were also analyzed: tahini with honey and spirulina (ingredients: spirulina, tahini, honey, and agave syrup), cocoa-praline spread with spirulina (ingredients: spirulina, tahini, grape syrup, honey, cocoa, and ground hazelnuts), and a cereal bar with lemon and spirulina (ingredients: date, oat flakes, almond, hemp protein, linseed, spirulina, coconut flour, and lemon butter). The two spreads were obtained from the same Greek manufacturer, while the cereal bar was manufactured in Bulgaria. All of these products were obtained from the Greek market.

### 2.2. Measurement of pH

The pH of all the tested products was measured either by inserting a pH meter electrode (CRISON micropH2001) directly into the product (tahini with honey, cocoa-praline), or by diluting 1:3 in sterile deionized water and mixing or pulverizing the food with a spatula to form a slurry (lemon cereal bar, tablets and powder). Three separate measurements were taken from each product and the mean was subsequently calculated.

### 2.3. Microbiological Analysis and Isolation of Bacteria

Five grams of the spirulina dietary supplements, and ten grams of the three spirulina-containing foods, were weighed into a sterile plastic bag with 45 mL and 90 mL of sterile diluent (quarter-strength Ringer’s solution, Lab M, Heywood, UK), respectively. The sample and diluent were homogenised in a laboratory paddle blender (Bag Mixer 400, Seward Ltd., Worthing, UK) for 60 s, then serially, decimally diluted in 9 mL aliquots of sterile diluent. For the spore counts, the homogenised sample (10 mL) was heated to 80 °C for ten minutes, and then cooled in a room-temperature water bath before further decimal dilutions were performed. The diluted sample was inoculated into agar plates. The media used, the inoculation technique, and the incubation of the plates are as presented in Table 1. All media used were sterilised at 121 °C for 15 min, with the exception of violet red bile agar, salmonella-shigella agar, xylose lysine desoxycholate agar and thiosulfate citrate bile salts sucrose agar, which were heated at 100 °C for 5 min, and Skimmed Milk Powder which was heated at 121 °C for 5 min. Following incubation of the plates and enumeration of the colonies, microbial counts were transformed to logarithms. Only colonies typical of the target organism according to descriptions provided by the media manufacturer and by the FDA Bacteriological Analytical Manual [12] were enumerated on selective media.

Fifty-five colonies were selected from different agar plates and were isolated by streaking onto fresh plates of the same agar media. Colonies were selected to represent the main colony morphologies observed. All isolates were examined microscopically (Carl Zeiss AXIO Lab.A1, Oberkochen, Germany) to determine the cell morphology and motility. These 55 isolates were identified using matrix-assisted laser desorption ionization-time of flight mass spectrometry (see Section 2.4). Selected plate count agar (PCA), and reinforced clostridial agar (RCA) plates from the colony counts were retained for 16S rRNA gene sequencing analysis (see Section 2.6).

### 2.4. Bacterial Identification Using Matrix-Assisted Laser Desorption Ionization-Time of Flight Mass Spectrometry

Selected bacterial isolates were identified using matrix-assisted laser desorption ionization-time of flight mass spectrometry (MALDI-TOF MS, Microflex LT model, Bruker-Daltonics, Billerica, MA, USA). The isolates were grown previously in brain heart infusion (Merck, Darmstadt, Germany) agar plates for 24 h at 30 °C. After incubation, one isolated colony was picked using a sterile inoculation loop and placed in a well of the surface of a MSP96 Bruker steel plate, and was left to dry at room temperature for 5 min. One microliter of matrix solution (HCCA, Bruker-Daltonics) was added on top of each sample and was left to dry for 5 min at room temperature in a laminar flow cabinet. Each batch of samples included a control Bruker BTS standard sample (a mass calibration standard showing a typical *Escherichia coli* DH5 alpha peptide and protein profile plus additional proteins). The isolates were compared with the commercial spectra reference library database provided by Bruker Daltonics MALDI Biotyper.

### 2.5. Detection of Cyanotoxins Using Enzyme-Linked Immunosorbent Assay (ELISA)

For microcystins (MCYSTs) analysis, all samples (0.5 g of each sample) were separately homogenized in 15 mL 75% (*v*/*v*) aqueous methanol [13]. They were treated with ultrasonication (15 W, 22.5 kHz), and were stirred overnight at room temperature (20–22 °C), followed by centrifugation at 13,000× *g* for 15 min. The extraction procedure was repeated three times. Supernatants were evaporated to dryness by vacuum centrifugation. Dried extracts were re-suspended in 15 mL deionized water, vigorously mixed, and sonicated for 15 min in an ice-cold ultrasound bath. C18 solid phase extraction (SPE) was performed as according to the manufacturer’s instructions (Waters GmbH, Eschborn, Germany) to purify and concentrate MCYSTs in the extracts.

For cylindrospermopsins (CYNs) and saxitoxins (STXs) analysis, all samples were extracted (24 h) with 5% formic acid in water, using 30 mL of extraction solvent [14], and then sonicated for 10 min. After sonication, the mixture was stirred for 30 min at room temperature, centrifuged for 10 min at 13,000× *g*, following which the supernatant was collected. All extracts were prepared for ELISA analysis by the removal of organic solvents, and re-suspension of the samples in phosphate-buffered saline (PBS) to adjust the pH to 6.5–7.5.

Microcystin (520011) (Eurofins/Abraxis, Warminster, PA, USA), Saxitoxin (52255B) (Eurofins/Abraxis, Warminster, PA, USA), and cylindrospermopsin (522011) (Eurofins/Abraxis, Warminster, PA, USA) microtiter Plate Kits were used to determine the presence of MCYSTs, STXs, and CYNs, respectively in all dietary samples. ELISA was performed according to the manufacturer’s directions using a multiwall Labtech LT-4500 microplate reader (Uckfield, UK) for the detection of the colorimetric product of antibody-conjugated enzymes.

Recovery experiments were carried out in quadruplicate for each cyanotoxin separately, by spiking 0.5 g of *Chlorella*-based dietary supplement (free of cyanotoxins, according to manifacturers’ quality measurements) with MCYST-LR, CYNs, and STXs standards (Eurofins/Abraxis, USA), at concentrations of 1, 2, and 15 μg g^−1^, from each standard. The matrix effect of MCYSTs analysis was checked by spiking 0.5 g of *Chlorella*-based dietary supplement with MCYST-LR standard (at three concentrations: 1, 2, and 15 μg g^−1^), and the response was compared to the extraction solvent of MCYSTs (75% methanol) spiked with the same amount of the MCYST-LR standard. The matrix effect of CYNs analysis was checked by spiking 0.5 g of *Chlorella*-based dietary supplement with CYNs standard (at three concentrations: 1, 2, and 15 μg g^−1^), and the response was compared to the extraction solvent of CYNs (5% formic acid in water) spiked with the same amount of CYNs standard. Additionally, the matrix effect of STXs analysis was checked by spiking 0.5 g of *Chlorella*-based dietary supplement with STXs standard (at three concentrations: 1, 2, and 15 μg g^−1^), and the response was compared to the extraction solvent of SXTs (5% formic acid in water) spiked with the same amount of STXs standard. Seventeen percent methanol was used as the final solvent in the MCYSTs analyses, which is known to produce no false-positives [15].

The estimated daily intake (EDI) is defined as the presumed daily exposure to, or consumption of a nutrient or chemical residue. The EDI of MCYSTs, through the consumption of spirulina products was calculated in the present study as follows [16,17]:

EDI = (C × Cons)/Bw (1)

where C is the concentration of MCYSTs in contaminated spirulina product, Cons stands for the daily average consumption of spirulina product as recommended by the producers, and Bw represents the body weight of the consumer (infants: 5 kg, children: 20 kg, and adults: 60 kg). EDI was then compared to the tolerable daily intake (TDI), which is an estimate of the amount of microcystins that can be ingested daily over a lifetime without appreciable health risk. TDIs for different age groups are as follows. infants (5 kg): 200 ng MCYSTs, children (20 kg): 800 ng MCYSTs, and adults (60 kg): 2400 ng MCYSTs [16,17].

### 2.6. Amplicon Sequencing Analysis

We analyzed the diversity of the V3-V4 region of the 16S rRNA gene following bulk DNA extraction from all colonies grown on agar plates and whole foods. For each product, three samples were prepared: the food itself, combined colonies from PCA plates (aerobic), and combined colonies from RCA plates (anaerobic). After scraping the agar plate colonies with 300 μL of MRD under aseptic conditions, the scraped material was collected in Eppendorf tubes. Each tube was hand-spun for 30–60 s, until the supernatant was clear, and then it was discarded. Around 0.3–0.4 μg of food sample was collected in Eppendorf tubes, and along with the cell pellets from the agar plates, were stored immediately at −80 °C until further processing.

Bulk DNA was isolated using the QIAGEN QIAamp DNA Mini Kit (Qiagen, Hilden, Germany), according to the manufacturer’s protocol. The primer pair S-D-Bact-0341-b-S-17 and S-D-Bact-115 0785-a-A-21 [18] targeting the V3–V4 regions of the bacterial 16S rRNA gene was used for PCR amplification, and sequenced on a MiSeq Illumina instrument (2 × 300 bp) at the MRDNA Ltd. (Shallowater, TX, USA) as according to their standard protocols. The sequencing data can be accessed in the Sequence Read Archive (https://www.ncbi.nlm.nih.gov/sra/ (accessed on 16 March 2023)) BioProject PRJNA926888. The raw 16S rRNA sequencing data were processed using the MOTHUR standard operating procedure (v.1.45.3) [19,20], and the OTUs at 97% cut-off similarity level were classified with the SILVA database release 138 [21,22]. The closest relatives of the resulting OTUs were identified by using a nucleotide blast (http://blast.ncbi.nlm.nih.gov (accessed on 16 March 2023)).

## 3. Results

### 3.1. pH Measurements

The measured pH of the five test samples were as follows (mean ± standard deviation, three replicates): spirulina powder 7.1 ± 0.0; spirulina tablets 7.2 ± 0.1; lemon cereal bar 4.9 ± 0.2; tahini with honey 5.4 ± 0.1; and cocoa-praline 7.1 ± 0.1.

### 3.2. Microbiological Analysis Using Culture-Dependent Techniques

The microbiological profiles of the foods and supplements as determined by analysis using culture techniques are presented in Table 2. Counts of viable microorganisms were generally low and did not exceed 3.2 log cfu/g. Endospore-forming bacteria made up a large proportion of the total count in the spirulina powder, the tahini with honey, and the cocoa-praline, but not in the spirulina tablets or the lemon cereal bars. The use of selective media did not result in the isolation of colonies in most cases, except for around 1000 cfu/g presumptive *Pseudomonas* spp. And yeasts and moulds in the spirulina powder, and about the same number of presumptive lactic acid bacteria in the tahini with honey. *Listeria* spp., coliforms (both with detection limit 2.0 log cfu/g), *Staphylococcus aureus*, and enterococci (both with detection limit 2.7 log cfu/g) were not detected in any samples. *Salmonella enterica* and *Vibrio* spp. were not detected in 25 g of the three food products nor in 5 g of the two supplements.

### 3.3. Microbiological Analysis by Amplicon Sequencing

The phyla Proteobacteria (Pseudomonadota), and Firmicutes (Bacillota), each represented about one-third of the detected bacteria, while a further 13.4% were in the phylum Actinobacteria (Actinomycetota) (Table 3). A total of nine different phyla were detected. Of the 39 different bacterial families detected, Bacillaceae were the most abundant (13.5%), followed by Rhodobacteraceae (9.5%), and Enterobacteriaceae (6.8%).

**Figure 1:**
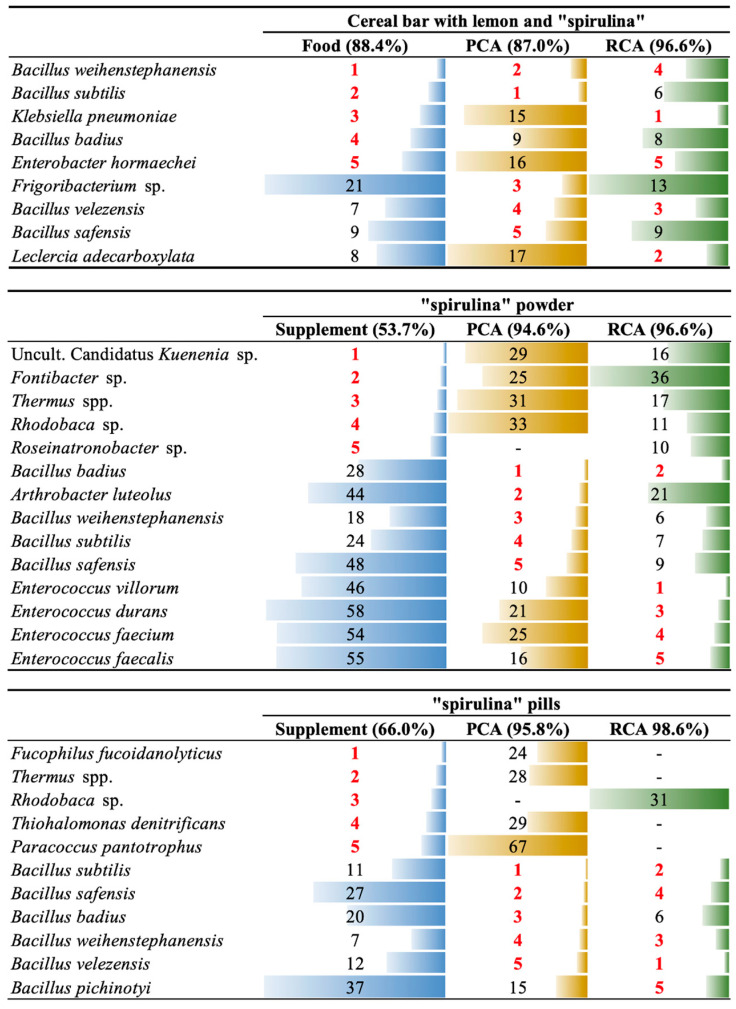
Rank abundance of the top five operational taxonomic units (OTUs, in red numbers) found in spirulina-containing foods and supplements, and in two culture media (plate count agar (PCA) incubated aerobically, and reinforced clostridial agar (RCA) incubated anaerobically, both at 30 °C). Percentage values indicate the cumulative relative abundance of the top five OTUs in each sample. Filled bars are proportional to the abundance ranking number (smallest bar = top of ranking).

In Figure 1, the main species detected in each individual sample for which both the product itself and the culture plates were analysed are presented. The tables give the five OTUs with the highest relative abundance as determined by the analysis of the product itself, by analysis of the aerobic culture plates (PCA), and by analysis of the anaerobic culture plates (RCA). OTUs were individual species or genera. In general, *Bacillus* spp., *Enterococcus* spp., and members of the Enterobacteriaceae were the most abundant colonies on the agar plates used for enumeration, and this was also the case with the cereal bar when analysed directly. However, analysis of the two supplements revealed a significant difference between the bacterial populations detected directly in the supplement, and the bacterial populations that grew on the enumeration plates. For two of the products, cocoa-praline and tahini with honey, direct analysis of the product was not possible. Identification of the bacteria on the agar plates from these products showed that *Bacillus* spp. (*B. weihenstephanensis, B. velezensis* and *B. subtilis*) was predominant in the cocoa-praline, while the main species in the tahini with honey were *Bacillus weihenstephanensis, Enterobacter hormaechei, Klebsiella pneumoniae*, and *Leclercia adecarboxylata.*

In addition to the bacteria listed in Figure 1 and in the preceding paragraph, species from the following genera were detected at a lower relative abundance in one or more samples as follows: *Acidobacterium, Alterococcus, Arthrobacter, Chelatococcus, Halomonas, Oceanicaulis, Rhodobacter, Rhodopirellula,* and *Weissella.*

### 3.4. Identification of Individual Isolates by MALDI-TOF

The isolates identified by the MALDI-Biotyper system, 55 in total, are presented in Table 4. The most widely distributed genera were *Bacillus*, which was isolated from all five products, *Enterococcus*, and *Micrococcus*.

### 3.5. Cyanotoxins

Results from the validation measurements showed that the recovery of MCYSTs, CYNs, and STXs from the spiked samples was 85 ± 2.2%, 87 ± 2.1%, and 91 ± 3.1%, respectively. The matrix effect was negligible for each cyanotoxin analysis (1.7 ± 0.5% for MCYSTs analysis, 1.9 ± 0.5% for CYNs analysis, and 2.1 ± 0.4% for STXs analysis, respectively).

The results of the cyanotoxin analysis are presented in Table 5. STXs and CYNs were not detected in any of the samples. All samples were contaminated with MCYSTs at concentrations ranging from 143 to 321 ng g^−1^. The highest concentrations were observed in the two spirulina dietary supplements (tablets and powder).

**Figure 2:**
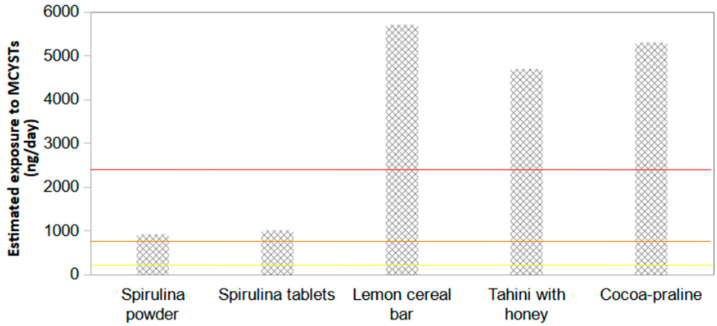
Microcystins (ng per day) estimated exposures from spirulina supplements and supplemented foods (spirulina powder, spirulina tablets, lemon cereal bar, tahini with honey, and cocoa-praline) in comparison to the maximum tolerable daily intakes calculated for infants, children, and adults. Tolerable daily intake of infants (5 kg): 200 ng MCYSTs (yellow line), children (20 kg): 800 ng MCYSTs (orange line), and adults (60 kg): 2400 ng MCYSTs (red line) [16,23].

Estimated daily MCYSTs exposure was 900 ng for spirulina powder, 1000 ng for spirulina tablets, 5700 ng for lemon cereal bar, 4700 ng for tahini with honey, and 5300 ng for cocoa-praline. The lemon cereal bar, tahini with cocoa and cocoa-praline samples exceeded the MCYSTs tolerate daily ingestion (TDI) set for infants, children, and adults. Meanwhile, the spirulina powder and tablets exceeded the MCYSTs TDI set for infants and children (Figure 2).

## 4. Discussion

The pH value of the five products was measured to determine whether it was likely to be a factor in the survival of bacteria in the products during storage. Three of the products had a pH close to neutral and two were mildly acidic, but not to the extent that the pH by itself was likely to affect the survival of most bacteria. The water activity was not measured, but all five products assessed were low-moisture, shelf-stable foods, so any safety issues with bacterial pathogens are likely to concern survival and cross-contamination, not growth in the products themselves.

The results of the amplicon sequencing analysis (Figure 1) and Section 3.3 indicate a fairly diverse bacterial population in the foods and supplements. For three products, the spirulina in the powder and tablet forms, and the lemon cereal bars, analysis was conducted on the product itself, and on agar plates used for the isolation of viable bacteria. For the tahini with honey and the cocoa-praline, analysis was conducted on the agar plates only, as no amplification was possible directly from these foods. These last two samples had a greatly reduced apparent microbial diversity compared to those in which the food itself was analysed, most probably due to the detection limit of the plate-counting method, and the ability of certain bacteria to grow on the media under the incubation conditions used. It can be observed that *Bacillus* spp. are predominant, which is to be expected given their ability to form endospores, which can survive the various heating and drying processes undergone by the products. In total, six species of *Bacillus* were detected, which did not include the foodborne pathogen *B. cereus*, but did include the closely related species *B. weihenstephanensis*.

The bacterial isolates identified by MALDI-TOF (Table 4) comprise a smaller range of species compared to those detected by amplicon sequencing. This is perhaps to be expected, as these isolates represent only those that were culturable in the isolation media used. In addition, as a few colonies representative of visually distinguishable phenotypes were selected, some species that had grown on the agar plates used for the initial enumeration may not have been isolated for identification. It is notable that the potential pathogen *B. cereus* was identified in four of the five samples. The *B. cereus* group consists of six closely related species, including *B. cereus* itself, the pathogen *B. anthracis*, the agricultural biocontrol agent *B. thuringiensis*, and *B*. *weihenstephanensis.* The latter is a relatively newly described species, first proposed in 1998 [24]. The members of the *B. cereus* group are extremely difficult to differentiate, both by 16S rRNA sequencing, and by protein profiling (MALDI-TOF) when standard commercially available libraries are used [25,26,27]. In the current study, *B. weihenstephanensis* was detected in all five products by amplicon sequencing, with particularly high relative abundances in the cocoa-praline and the tahini with honey, and low relative abundances in the spirulina powder and tablets. Identification of the isolated colonies by MALDI-TOF showed that *B. cereus* was present in all the samples apart from the spirulina powder, with multiple colonies isolated from the cocoa-praline and tahini with honey. It therefore seems likely (but is not proven) that the bacteria identified as *B. weihenstephanensis* by amplicon sequencing and as *B. cereus* by MALDI-TOF, are in fact the same species. Which of the two identifications is correct has not been determined and may not be of great importance for the safety of the products, as some isolates of *B. weihenstephanensis* have been shown to produce some or all of the same enterotoxins as *B. cereus* [28,29].

The presence of enterotoxin-producing *Bacillus* spp. in these products is potentially hazardous. Foodborne illness caused by *B. cereus* is normally a result of the production of the enterotoxin in the food prior to consumption [30]. In the products examined in the current work, the low water activity would prevent the growth of the bacterium to hazardous levels. However, if the products were rehydrated, or added to other foods (e.g., the cocoa-praline used as an ingredient in desserts), the environment could become favorable for growth and toxin production. *B. cereus* may also cause diarrheal disease, in this case by colonizing the gut and producing a different enterotoxin [30]. This is of particular concern in spirulina products, as they are sometimes given as a supplement to patients with other diseases. These patients may be more susceptible to enteric illnesses if they are immunocompromised or have an imbalance in their normal gut microbiome due to illness or antibiotic therapy, which renders it less resistant to colonization by newly introduced bacteria. The origin of the *B. cereus* in the three food products has not been established. *Bacillus* spp., and specifically *B. cereus*, are frequently isolated from some of the Ingredients of the three products examined, such as honey, cereals, and cocoa [31,32,33], and so may not be related to the addition of spirulina to the products.

Other bacteria detected in the examined products are potential human pathogens. *Klebsiella pneumoniae* is an opportunistic pathogen that can cause bacteremia, pneumonia, urinary tract infections, and liver abscesses [34,35]. It is not normally regarded as a foodborne pathogen, but evidence is emerging that food may sometimes act as a vector of the disease. Enteric carriage of *K. pneumoniae* has been shown to correlate with an increased incidence of pyogenic liver abscesses in Taiwan [36], and food can act as a vector for colonization of the large intestine. Epidemiological and serological evidence linking cases of *K. pneumoniae* bacteremia with contaminated food have been obtained from a patient in the United States who became sick after eating a contaminated beefburger [37], and from a nosocomial outbreak in Spain [38]. Apart from the immediate danger of infection, *K. pneumoniae* has a high incidence of antibiotic resistance, often as a result of mobile genetic elements that can potentially be transferred to other bacteria in the gut, including potential pathogens [35]. The presence of *K. pneumoniae* in food therefore presents a potential risk that is difficult to quantify with the currently available data.

*Leclercia adecarboxylata* has been recognized as an emerging opportunistic pathogen [39] that has been implicated in bacteremia, endocarditis, peritonitis, urinary tract infections, and pneumonia. Patients infected are mainly, but not exclusively, immunocompromised. It is a member of the family Enterobacteriaceae, and is widely distributed in nature. The epidemiology and mechanism of *L. adecarboxylata* infection is poorly understood, in part because the organism can be easily misidentified as *Escherichia coli* in laboratories relying on conventional microbiological techniques, but there is evidence supporting translocation from the gut as one route [40]. Another opportunistic pathogen that was detected in a relatively smaller abundance in the lemon cereal bar and in the tahini with honey was *Weissella confusa*. These *Lactobacillus*-like bacteria can cause bacteremia, endocarditis, and abscesses, and can migrate from the gut into the blood if there is some disruption to the gut mucosa, or to the normal microbial population of the intestine. They are inherently resistant to vancomycin, so treatment of a patient with this drug may exert a positive selective pressure on the bacterium [41,42].

The three potential opportunistic pathogens described above occupy a variety of microbiological niches, and are fairly widely distributed in nature [34,39,41]. They were detected in the cereal bar and in the tahini with honey. The source of contamination of these products cannot be determined as several of the ingredients are possible vectors. *Klebsiella pneumoniae* has a relatively high incidence in food, as evidenced by surveys of various food types. In a survey in Singapore, 15% of ready-to-eat cooked foods from markets contained this bacterium [34].

Many of the bacteria detected, mostly in a low relative abundance, were those associated with aquatic and marine environments, and so were most likely present in the pools used for the production of the spirulina. Examples include bacteria from the genera *Thermus, Oceanicaulis, Fontibacter, Rhodopirellula, Halomonas, Rhodobaca, Rhodobacter, Roseinatronobacter,* and *Thiohalomonas.*

The apparent discrepancy in this study between 16S rRNA sequencing and MALDI-TOF as a means of identification has broader implications, and the two methods should be seen as complementary, which when combined, reveal a wider picture. Although the samples analyzed were not exactly the same (individual colonies vs. whole plate or product sample), the fact that so few bacteria in common were found, together with the possible confusion of *B. cereus* and *B. weihenstephanensis* discussed above, calls into question the reliability of one or both of the methods. Both methods of identification are only as good as the databases that were used to generate an identification from the analytical data. The MALDI-TOF method is newer, and so the commercial databases commonly used are less comprehensive [27]. Despite the fact that that the amplicon sequencing analysis of the V3-V4 regions is considered an acceptable proxy for the taxonomic affiliation of the whole gene and the subsequent taxonomic affiliation [43,44], it lacks reliability in distinguishing closely related bacterial species. In the future, whole genome sequencing or metagenome-assembled genomes are to be used for distinguishing between the different species in the *B. cereus* group in similar samples.

Commercial *Arthrospira* is often grown in outdoor, industrial ponds designed to selectively maintain near monocultures of *Arthrospira* and avoid the growth of toxic cyanobacteria. However, maintenance problems can occur, and cyanotoxins have been detected in some spirulina products, which indicates that industrial ponds sometimes become contaminated with toxic cyanobacteria species. According to our results, toxic cyanobacterial species were not identified, but significant concentrations of cyanotoxins (MCYSTs) were detected in spirulina dietary supplements and supplemented foods. On a research level, several measurements of cyanotoxins in spirulina products have been carried out [45,46,47]. Del Olmo-Iruela et al. [48] determined seven cyanotoxins in spirulina supplements, belonging to three different classes. Among these classes were MCYSTs, which were found at concentration levels higher than 5 mg kg^−1^. Cyanotoxins, under the form of six MCYSTs congeners, were also detected in nine out of the thirty-five analyzed algal food supplements from the Belgian market, and contaminated products contained total MCYSTs of concentrations between 238.45 and 5645.33 µg kg^−1^, according to measurements of Van Hassel et al. [49]. At least 12 different species of cyanobacteria have been proven to produce cyanotoxins [50]. None of the known species producing cyanotoxins were identified by 16S rRNA sequencing or MALDI-TOF, according to our results. Papadimitriou et al. [51] also found spirulina supplements positive for MCYSTs, although some of them were negative to the presence of cyanobacteria. The authors explained that this could be due to methodological issues, concerning molecular methods including 16S rRNA sequencing. An alternative explanation for the presence of MCYSTs without the presence of cyanobacteria would be the possible contamination of spirulina products with extracellular cyanotoxins during the various stages of the production process. Most MCYSTs originate from cyanobacterial species, which can coexist in the same habitat with spirulina [52]. The majority of MCYSTs are intracellular toxins, however cyanobacterial cells that are lyzed or damaged release large amounts of toxins into the water (extracellular toxins) [53]. The extracellular MCYSTs are highly stable metabolites which can withstand the production process and contaminate the final spirulina product.

According to TDI guidelines [54,55], and MCΥSTs exposures from spirulina dietary products examined in the current study, the lemon cereal bar, tahini with cocoa and cocoa-praline supplements could pose risks concerning adult, children, and infant health. However, the small size of the samples tested, as well as the lack of toxicological testing, mean that it is impossible to accurately determine the risk to human health following the consumption of spirulina products used in the current study. Several factors must be considered when assessing the risk of consuming spirulina products contaminated with MCYSTs. Among them are the health of the people who consume the products, if they have pre-existing health conditions that could contribute to the risk, and the short-term or long-term use of these products. Also, it is important to consider that the levels of MCYSTs in a given brand name can vary extensively from batch to batch [55].

When people are exposed to cyanotoxins, adverse health effects may range from a mild skin rash to serious illness [56]. Acute health effects in humans include abdominal pain, headache, sore throat, vomiting and nausea, dry cough, diarrhea, blistering around the mouth, and pneumonia [57]. Chronic MCYSTs exposure through drinking water has also been linked to various adverse health effects, such as increased incidences of liver cancer, and other chronic liver ailments [58]. Although the most common routes of human exposure to MCYSTs are through ingestion or contact with water containing toxic cyanobacteria and cyanotoxins, in 2018, a case of microcystin poisoning after consumption of dietary supplements was recorded in the literature [59]. A lung cancer patient self-medicated themselves with natural products, which led to them beingdiagnosed with acute toxic hepatitis. The patient was taking nutritional supplements, including *Chlorella*, and 1.08 μg of MCYSTs per gram of *Chlorella* biomass was measured in the supplement, which may have contributed to the acute hepatitis in this patient. In conclusion, the quality of the commercial cultures used to produce algal dietary supplements requires a high level of quality control and assurance to avoid MCYSTs contamination and possible health risks [60].

## 5. Conclusions

Spirulina supplements and spirulina-containing foods may be contaminated with potentially pathogenic bacteria. This is of particular concern, as these supplements are often given to immunocompromised patients, who may be more susceptible to foodborne infections even from low levels of contamination. In addition, although the foods examined would be unlikely to support the growth of pathogenic bacteria to dangerous levels for healthy individuals, they provide an entry vehicle into the domestic environment, and a possible vector for cross-contamination to other foods which may allow proliferation of the bacteria. All of the products examined were contaminated with microcystins at a level which could, at least, contribute a significant proportion of the recommended safe intake of the consumer, and in some cases cause the limit to be exceeded even with no other dietary sources. Greater attention needs to be given to the safety of spirulina products, particularly the use of open ponds which are subject to external contamination and may allow the growth of other, toxin-producing, species of cyanobacteria and other potentially harmful bacteria.

## Figures and Tables

**Table 1:** Target organism, media, inoculation technique, and incubation used to identify specific microbial populations in spirulina samples.

Target Organisms	Plating Medium	Inoculation Technique	Incubation
Mesophilic microorganisms & *Bacillus* spores	Plate count agar (PCA) ^1^	Surface spread, quarter plate, 20 μL	30 °C, 72 h
*Clostridium* & *Clostridium* spores	Reinforced Clostridial Agar (RCA) ^1^	Surface spread, quarter plate, 20 μL	30 °C, 72 h
Lactic acid bacteria	De Man, Rogosa and Sharpe (MRSA)^1^	Surface spread, quarter plate, 20 μL	30 °C, 72 h, candle jars
Thermotolerant enterococci & streptococci	KF streptococcus medium (KF) ^2^	Surface spread, quarter plate, 20 μL	37 °C, 4 h then 44 °C, 44 h.
*Staphylococcus aureus*	Mannitol Salt Agar (MSA) ^1^	Surface spread, quarter plate, 20 μL	37 °C, 48 h
Yeasts and moulds	Yeast glucose chloramphenicol agar (YGC) ^3^	Surface spread, 100 μL	25 °C, 3–5 days
*Listeria* spp.	Oxford agar ^1^	Surface spread, 500 μL	30 °C, 48 h
Coliforms	Violet red bile agar (VRBA) ^1^	Pour plate, 1 mL, with overlay	37 °C, 48 h
*Pseudomonas* spp.	Cetrimide Agar medium ^4^ + 10 mL glycerol ^3^	Surface spread, quarter plate, 20 μL	37 °C, 48 h
*Vibrio* spp.	- Alkaline Peptone water ^5^ (Enrichment broth) - Thiosulfate Citrate Bile Salts Sucrose Agar ^3^ (TCBS)	10 g of sample in 90 mL Plates were streaked with isolation technique	37 °C, 24 h 37 °C, 24 h
*Salmonella* spp.	- Skimmed Milk Powder ^5^ (Pre-enrichment broth) - Tetrathionate Broth & Selenite broth ^5^ (Enrichment broths) - Xylose Lysine Desoxycholate Agar (XLD) & Salmonella-Shigella Agar ^5^ (SS) (Isolation on selective agars)	10 g of sample in 90 mL pre-enrichment broth 1 mL of pre-enrichment broth in 10 mL enrichment broths Plates were streaked with isolation technique	37 °C, 24 h 37 °C, 24 h 37 °C, 24 h

^1^ Lab M, Heywood, UK. ^2^ Panreac Quimica SA, Barcelona, Spain. ^3^ Merck, Darmstadt, Germany ^4^ Scharlau Chemie SA, Barcelona, Spain ^5^ Oxoid, Basingstoke, UK.

**Table 2:** Spoilage microorganisms in spirulina supplements and spirulina-containing foods as determined by plate counting.

Analysis	Powder	Tablets	Lemon Cereal Bar	Tahini with Honey	Cocoa-Praline
	log cfu/g ± Standard Deviation				
Aerobic plate count	3.2 ± 0.17	1.9 ± 0.25	2.2 ± 0.06	3.1 ± 0.11	2.5 ± 0.18
Anaerobic plate count	2.7 ± 0.43	<1.0	<1.0	3.1 ± 0.01	2.1 ± 0.12
Aerobic spore count	2.6 ± 0.41	1.7 ± 0.12	1.7 ± 0.14	2.7 ± 0.51	<2.0
Anaerobic spore count	2.7 ± 0.81	<1.0	<1.0	2.1 ± 0.12	<2.0
*Pseudomonas* spp.	2.8 ± 0.36	<2.7	<2.7	<2.7	<2.7
Lactic acid bacteria	<2.7	<2.7	<2.7	3.1 ± 0.12	<2.7
Yeasts and Moulds	3.0 ± 0.28	<2.7	<2.7	<2.7	<2.7

**Table 3:** Relative abundance (cumulative across all samples including both foods and culture plates) of the main bacterial phyla and families as determined by amplicon sequencing.

Taxonomic Level	Name	Relative Abundance (%)
Phylum	Proteobacteria Firmicutes Actinobacteria	33.8 32.4 13.5
Family	Bacillaceae Rhodobacteraceae Enterobacteriaceae Enterococcaceae Lactobacillaceae Microbacteriaceae Micrococcaceae	13.5 9.5 6.8 5.4 5.4 4.1 4.1

**Table 4:** Identification of the individual isolates (from the agar plates used in the enumeration of the bacteria) by MALDI-TOF.

Product	Isolation Medium	Number of Colonies	Species Identified
Spirulina powder	PCA, RCA, KF PCA PCA PCA MSA	6 4 1 1 1	*Enterococcus faecium* *Bacillus badius* *Bacillus subtilis* *Bacillus pumilus* *Bacillus mojavensis*
Spirulina tablets	SS XLD XLD TCBS PCA Oxford TCBS RCA RCA	1 1 1 1 1 1 1 1 1	*Enterobacter cloacae* *Enterobacter kobei* *Enterobacter asburiae* *Enterococcus faecium* *Micrococcus luteus* *Bacillus cereus* *Bacillus pumilus* *Curtobacterium albidum* *Pseudomonas synxantha*
Lemon cereal bar	PCA PCA, RCA PCA PCA PCA PCA PCA PCA	3 2 1 1 1 1 1 1	*Staphylococcus xylosus* *Staphylococcus hominis* *Micrococcus luteus* *Bacillus cereus* *Bacillus subtilis* *Bacillus mojavensis* *Bacillus altitudinis* *Pseudomonas rhodesiae*
Tahini with honey	Oxford, PCA, RCA PCA, RCA MRS, RCA MSA PCA PCA PCA	4 3 2 1 1 1 1	*Bacillus cereus* *Leucanostoc mesenteroides* *Bacillus subtilis* *Bacillus atrophaeus* *Bacillus vallismortis* *Brevibacterium casei* *Micrococcus luteus*
Cocoa-praline	PCA, RCA, Oxford MSA, Oxford PCA	6 2 1	*Bacillus cereus* *Bacillus licheniformis* *Enterococcus faecium*

**Table 5:** Cyanotoxins detected in spirulina dietary supplements and supplemented foods.

	MCYST	CYN	STX
Product	ng g^−1^		
Spirulina powder	285.8	<0.040	<0.015
Spirulina tablets	321.3	<0.040	<0.015
Lemon cereal bar	143.3	<0.040	<0.015
Tahini with honey	188.1	<0.040	<0.015
Cocoa-praline	212.2	<0.040	<0.015

## Data Availability

The sequencing data can be accessed in the Sequence Read Archive (https://www.ncbi.nlm.nih.gov/sra/ (accessed on 19 April 2023)) BioProject PRJNA926888.

## References

[1] Lafarga T., Fernández-Sevilla J.M., González-López C., Acién-Fernández F.G. (2020). *Spirulina* for the food and functional food industries. Food Res. Int..

[2] Muys M., Sui Y., Schwaiger B., Lesueur C., Vandenheuvel D., Vermeir P., Vlaeminck S.E. (2019). High variability in nutritional value and safety of commercially available Chlorella and Spirulina biomass indicates the need for smart production strategies. Bioresour. Tech..

[3] Wan D., Wu Q., Kuča K., Gupta R.C. (2016). Spirulina. Nutraceuticals: Efficacy, Safety and Toxicity.

[4] Vonshak A., Tomaselli L., Whitton B.A., Potts M. (2000). Arthrospira (*Spirulina*): Systematics and EcophysioIogy. The Ecology of Cyanobacteria.

[5] Masojídek J., Torzillo G. (2014). Mass Cultivation of Freshwater Microalgae. Reference Module in Earth Systems and Environmental Sciences [Online Collection].

[6] Borowitzka M.A., Levine I.A., Fleurence J. (2018). Microalgae in medicine and human health: A historical perspective. Microalgae in Health and Disease Prevention.

[7] Lee E.H., Park J.-E., Choi Y.-J., Huh K.-B., Kim W.-Y. (2008). A randomised study to establish the effects of spirulina in type 2 diabetes mellitus patients. Nutr. Res. Pract..

[8] Cingi C., Conk-Dalay M., Cakli H., Bal C. (2008). The effects of spirulina on allergic rhinitis. Eur. Arch. Otorhinolaryngol..

[9] Mao T.K., Van de Water J., Gershwin M.E. (2005). Effects of a Spirulina-based dietary supplement on cytokine production from allergic rhinitis patients. J. Med. Food.

[10] Hoekstra D.T., Volschenk H., Collins M., McMaster L.D. (2011). An investigation of *Clostridium* species present in nutraceutical preparations of *Arthrospira platensis* (*Spirulina*) for human consumption. J. Appl. Phycol..

[11] Vardaka E., Kormas K.A., Katsiapi M., Genitsaris S., Moustaka-Gouni M. (2016). Molecular diversity of bacteria in commercially available “Spirulina” food supplements. PeerJ.

[12] Anon Bacteriological Analytical Manual (BAM). US Food and Drug Administration. https://www.fda.gov/food/laboratory-methods-food/bacteriological-analytical-manual-bam.

[13] Fastner J., Flieger I., Neumann U. (1998). Optimized extraction of microcystins from field samples—A comparison of different solvents and procedures. Water Res..

[14] Törökné A., Asztalos M., Bánkiné M., Bickel H., Borbély G., Carmeli S., Codd G.A., Fastner J., Huang Q., Humpage A. (2004). Interlaboratory comparison trial on cylindrospermopsin measurement. Anal. Biochem..

[15] Metcalf J.S., Bell S.G., Codd G.A. (2001). Colorimetric immuno-protein phosphatase inhibition assay for specific detection of microcystins and nodularins of cyanobacteria. Appl. Environ. Microbiol..

[16] Dietrich D., Hoeger S. (2005). Guidance values for microcystins in water and cyanobacterial supplement products (blue-green algal supplements): A reasonable or misguided approach?. Toxicol. Appl. Pharmacol..

[17] Humpage A.R., Falconer I.R. (2003). Oral toxicity of the cyanobacterial toxin cylindrospermopsin in male Swiss albino mice: Determination of no observed adverse effect level for deriving a drinking water guideline value. Environ. Toxicol..

[18] Klindworth A., Pruesse E., Schweer T., Peplies J., Quast C., Horn M., Glöckner F.O. (2013). Evaluation of General 16S Ribosomal RNA Gene PCR Primers for Classical and Next-Generation Sequencing-Based Diversity Studies. Nucleic Acids Res..

[19] Schloss P.D., Westcott S.L., Ryabin T., Hall J.R., Hartmann M., Hollister E.B., Lesniewski R.A., Oakley B.B., Parks D.H., Robinson C.J. (2009). Introducing Mothur: Open-Source, Platform-Independent, Community-Supported Software for Describing and Comparing Microbial Communities. Appl. Environ. Microbiol..

[20] Schloss P.D., Gevers D., Westcott S.L. (2011). Reducing the effects of PCR amplification and sequencing artifacts on 16S rRNA-based studies. PLoS ONE.

[21] Quast C., Pruesse E., Yilmaz P., Gerken J., Schweer T., Yarza P., Peplies J., Glöckner F.O. (2013). The SILVA ribosomal RNA gene database project: Improved data processing and web-based tools. Nucleic Acids Res..

[22] Yilmaz P., Parfrey L.W., Yarza P., Gerken J., Pruesse E., Quast C., Schweer T., Peplies J., Ludwig W., Glöckner F.O. (2014). The SILVA and “All-species Living Tree Project (LTP)” taxonomic frameworks. Nucleic Acid Res..

[23] Fromme H., Köhler A., Krause R., Führling D. (2000). Occurrence of cyanobacterial toxins—Microcystins and anatoxin-a—In Berlin water bodies with implications to human health and regulations. Environ. Toxicol..

[24] Lechner S., Mayr R., Francis K.P., Prüss B.M., Kaplan T., Wiessner-Gunkel E., Stewart G.S., Scherer S. (1998). *Bacillus weihenstephanensis* sp. nov. is a new psychrotolerant species of the *Bacillus cereus* group. Int. J. Syst. Bacteriol..

[25] Ha M., Jo H.-J., Choi E.-K., Kim Y., Kim J., Cho H.-J. (2019). Reliable identification of *Bacillus cereus* group species using low mass biomarkers by MALDI-TOF MS. J. Microbiol. Biothechnol..

[26] Manzulli V., Rondinone V., Buchicchio A., Serrecchia L., Cipolletta D., Fasanella A., Parisi A., Difato L., Iatarola M., Aceti A. (2021). Discrimination of *Bacillus cereus* group members by MALDI-TOF mass spectrometry. Microorganisms.

[27] Pauker V.I., Thoma B.R., Grass G., Bleichert P., Hanczaruk M., Zöller L., Zange S. (2018). Improved discrimination of *Bacillus anthracis* from closely related species in the *Bacillus cereus sensu lato* group based on matrix-assisted laser desorption ionization—Time of flight mass spectrometry. J. Clin. Microbiol..

[28] Stenfors L.P., Mayr R., Scherer S., Granum P.E. (2002). Pathogenic potential of fifty *Bacillus weihenstephanensis* strains. FEMS Microbiol. Lett..

[29] Thorsen L., Hansen B.M., Nielsen K.F., Hendriksen N.B., Phipps R.K., Budde B.B. (2006). Characterization of emetic *Bacillus weihenstephanensis*, a new cerelulide-producing bacterium. Appl. Environ. Microbiol..

[30] Stenfors Arnesen L.P., Fagerlund A., Granum P.E. (2008). From soil to gut: *Bacillus cereus* and its food poisoning toxins. FEMS Microbiol. Rev..

[31] Pomastowski P., Złoch M., Rodzik A., Ligor M., Kostrzewa M., Buszewski B. (2019). Analysis of bacteria associated with honeys of different geographical and botanical origin using two different identification approaches: MALDI-TOF MS and 16S rDNA PCR technique. PLoS ONE.

[32] Daczkowska-Kozon E.G., Bednarczyk A., Biba M., Repich K. (2009). Bacteria of *Bacillus cereus* group in cereals at retail. Pol. J. Food Nutr. Sci..

[33] Eijlander R.T., Breitenwieser F., de Groot R., Hoornstra E., Kamphuis H., Kokken M., Kuijpers A., de Mello I.I.G., de Rijdt G.V., Vadier C. (2020). Enumeration and identification of bacterial spores in cocoa powders. J. Food Prot..

[34] Hartantyo S.H.P., Chau M.L., Hoh T.H., Yap M., Yi T., Cao D.Y.H., Gutiérrez R.A., Ng L.C. (2020). Foodborne *Klebsiella pneumoniae*: Virulence potential, antibiotic resistance, and risks to food safety. J. Food Prot..

[35] Theocharidi N.A., Balta I., Houhoula D., Tsantes A.G., Lalliotis G.P., Polydera A.C., Stamatis H., Halvatsiotis P. (2022). High prevalence of *Klebsiella pneumoniae* in Greek meat products: Detection of virulence and antimicrobial resistance genes by molecular techniques. Foods.

[36] Fung C.P., Lin Y.T., Lin J.C., Chen T.L., Yey K.M., Chang F.Y., Chuang H.C., Wu H.S., Tseng C.P., Siu L.K. (2012). *Klebsiella pneumoniae* in gastrointestinal tract and pyogenic liver disease. Emerg. Infect. Dis..

[37] Sabota J.M., Hoppes W.L., Ziegler J.R., DuPont H., Mathewson J., Rutecki G.W. (1998). A new variant of food poisoning: Enteroinvasive *Klebsiella pneumoniae* and *Escherichia coli* sepsis from a contaminated hamburger. Am. J. Gastroenterol..

[38] Calbo E., Freixas N., Xercavins M., Riera M., Nicolás C., Monistrol O., Solé Mdel M., Sala M.R., Vila J., Garau J. (2011). Foodborne nosocomial outbreak of SHV1 and CTX-M-15-producing *Klebsiella pneumoniae*: Epidemiology and control. Clin. Infect. Dis..

[39] Zayet S., Lang S., Garnier P., Pierron A., Plantin J., Toko L., Royer P.-Y., Villemain M., Klopfenstein T., Gendrin V. (2021). Leclercia adecarboxylata as emerging pathogen in human infections: Clinical features and antimicrobial susceptibility testing. Pathogens.

[40] Al Shuhoumi M.A., Al Mhrooqi A., Al Rashdi A., Kumar R., Al Jabri A., Al Kalbani A., Al Jardani A. (2023). First clinical case of VIM-1-producing *Leclercia adecarboxylata*: A case report and literature review. Med. Microecol..

[41] Kamboj K., Vasquez A., Balada-Lioasat J.-M. (2015). Identification and significance of *Weisella* species infections. Front. Microbiol..

[42] Spiegelhauer M.R., Yusibova M., Rasmussen I.K.B., Fuglsang K.A., Thomsen K., Andersen L.P. (2020). A case report of polymicrobial bacteremia with *Weissella confusa* and a comparison of previous treatment for successful recovery with a review of the literature. Access Microbiol..

[43] García-López R., Cornejo-Granados F., Lopez-Zavala A.A., Sánchez-López F., Cota-Huízar A., Sotelo-Mundo R.R., Guerrero A., Mendoza-Vargas A., Gómez-Gil B., Ochoa-Leyva A. (2020). Doing more with less: A comparison of 16S hypervariable regions in search of defining the shrimp microbiota. Microorganisms.

[44] Sirichoat A., Sankuntaw N., Engchanil C., Buppasiri P., Faksri K., Namwat W., Chantratita W., Lulitanond V. (2021). Comparison of different hypervariable regions of 16S rRNA for taxonomic profiling of vaginal microbiota using next-generation sequencing. Arch. Microbiol..

[45] Saker M.L., Jungblut A.D., Neilan B.A., Rawn D.F., Vasconcelos V.M. (2005). Detection of microcystin synthetase genes in health food supplements containing the freshwater cyanobacterium Aphanizomenon flos-aquae. Toxicon.

[46] Marsan D.W., Conrad S.M., Stutts W.L., Parker C.H., Deeds J.R. (2018). Evaluation of microcystin contamination in blue-green algal dietary supplements using a protein phosphatase inhibition-based test kit. Heliyon.

[47] Miller T.R., Xiong A., Deeds J.R., Stutts W.L., Samdal I.A., Løvberg K.E., Miles C.O. (2020). Microcystin toxins at potentially hazardous levels in algal dietary supplements revealed by a combination of bioassay, immunoassay, and mass spectrometric methods. J. Agric. Food Chem..

[48] Del Olmo Iruela M., Del Mar Aparicio-Muriana M., Lara F.J., Garcia-Campaña A.M. (2022). Determination of multiclass cyanotoxins in spirulina-based dietary supplements using a SLE-Tandem-SPE procedure followed by HILIC-MS/MS. Biol. Life Sci. Forum.

[49] Van Hassel W.H.R., Ahn A.C., Huybrechts B., Masquelier J., Wilmotte A., Andjelkovic M. (2022). LC-MS/MS validation and quantification of cyanotoxins in algal food supplements from the Belgium market and their molecular origins. Toxins.

[50] Okamoto K., Fleming L.E., Wexler P. (2005). Algae. Encyclopedia of Toxicology.

[51] Papadimitriou T., Kormas K., Vardaka E. (2021). Cyanotoxin contamination in commercial Spirulina food supplements. J. Consum. Prot. Food Saf..

[52] Singh S., Kate B.N., Banerjee U.C. (2005). Bioactive compounds from cyanobacteria and microalgae: An overview. Crit. Rev. Biotechnol..

[53] Cousins L.T., Bealing D.J., James H.A., Sutton A. (1996). Biodegradation of microcystin-LR by indigenous mixed bacterial populations. Water Res..

[54] WHO (1998). Cyanobacterial Toxins: Microcystin-LR, Guidelines for Drinking-Water Quality.

[55] Gilroy D.J., Kauffman K.W., Hall R.A., Huang X., Chu F.S. (2000). Assessing potential health risks from microcystin toxins in blue-green algae dietary supplements. Environ. Health Perspect..

[56] Codd G.A., Metcalf J.S., Beattie K.A. (1999). Retention of *Microcystis aeruginosa* and microcystin by salad lettuce (*Lactuca sativa*) after spray irrigation with water containing cyanobacteria. Toxicon.

[57] Fawell J.K., Mitchell R.E., Everett D.J., Hill R.E. (1999). The toxicity of cyanobacterial toxins in the mouse: I microcystin-LR. Hum. Exp. Toxicol..

[58] Svirčev Z., Lalić D., Bojadžija Savić G., Tokodi N., Drobac Backović D., Chen L., Meriluoto J., Codd G.A. (2019). Global geographical and historical overview of cyanotoxin distribution and cyanobacterial poisonings. Arch. Toxicol..

[59] Costa M.L., Rodrigues J.A., Azevedo J., Vasconcelos V., Eiras E., Campos M.G. (2018). Hepatotoxicity induced by paclitaxel interaction with turmeric in association with a microcystin from a contaminated dietary supplement. Toxicon.

[60] Grobbelaar J.U. (2003). Quality control and assurance: Crucial for the sustainability of the applied phycology industry. J. Appl. Phycol..

